# Pterostilbene Confers Protection against Diquat-Induced Intestinal Damage with Potential Regulation of Redox Status and Ferroptosis in Broiler Chickens

**DOI:** 10.1155/2023/8258354

**Published:** 2023-01-24

**Authors:** Yanan Chen, Hao Zhang, Yue Li, Tian Wang

**Affiliations:** ^1^College of Animal Science & Technology, Nanjing Agricultural University, Nanjing, Jiangsu 210095, China; ^2^Institute of Animal Science, Jiangsu Academy of Agricultural Sciences, Nanjing 210014, China

## Abstract

Oxidative stress causes damage to macromolecules, including proteins, DNA, and lipid, and has been recognized as a crucial driver of the onset and progression of several intestinal disorders. Pterostilbene, one of the natural antioxidants, has attracted considerable attention owing to its multiple biological activities. In the present study, we established an oxidative stress model in broiler chickens via injection with diquat to investigate whether pterostilbene could attenuate diquat-induced intestinal damage and reveal the underlying mechanisms. We found that diquat-induced decreases in the activities of superoxide dismutase and glutathione peroxidase and the level of reduced glutathione and the increase in hydrogen peroxide content in plasma and jejunum were significantly alleviated by pterostilbene (*P* < 0.05). Pterostilbene supplementation also decreased intestinal permeability and jejunal apoptosis rate, improved jejunal villus height and the ratio of villus height to crypt depth, and promoted the transcription and translation of jejunal tight junction proteins occludin and zona occludens 1 in diquat-challenged broilers (*P* < 0.05). Furthermore, pterostilbene reversed diquat-induced mitochondrial injury in the jejunum, as indicated by the decreased reactive oxygen species level and elevated activities of superoxide dismutase 2 and mitochondrial respiratory complexes (*P* < 0.05). Importantly, administering pterostilbene maintained iron homeostasis, inhibited lipid peroxidation, and regulated the expression of the markers of ferroptosis in the jejunum of diquat-exposed broilers (*P* < 0.05). The nuclear factor erythroid 2-related factor 2 signaling pathway in the jejunum of diquat-exposed broilers was also activated by pterostilbene (*P* < 0.05). In conclusion, our study provides evidence that pterostilbene alleviates diquat-induced intestinal mucosa injury and barrier dysfunction by strengthening antioxidant capacity and regulating ferroptosis of broiler chickens.

## 1. Introduction

Oxidative stress is considered to be the result of a disturbance in the system's equilibrium where prooxidants overwhelm the antioxidant defense mechanisms [1]. This imbalance can induce oxidative modification of cellular macromolecules, cellular dysfunction, and death [2]. Currently, oxidative stress has been implicated in various pathological conditions, including intestinal disorders [3–5]. The small intestine is an important organ for the health and survival of animals. It not only plays a critical role in nutrient digestion and absorption but also provides a powerful barrier against pathogens and noxious agents [6]. All these actions involve the generation and clearance of free radicals, making the small intestine more vulnerable to oxidative stress than other organs [7]. The occurrence of oxidative stress in the small intestine can disrupt barrier defense, increase bacterial translocation, and evoke inflammatory response and necroptosis, even leading to the failure of the intestinal distal organs [8–10]. Therefore, strategies protecting the small intestine against oxidative damage are urgently sought.

Ferroptosis is an emerging form of regulated cell death with characteristics of ultrastructural alternations, including vanished mitochondria crista and condensed and ruptured mitochondrial membrane [11]. These features distinguish ferroptosis from other molecular programs controlling the demise of cells. The biochemical mechanism underlying ferroptosis is the iron-catalyzed accumulation of lipid reactive oxygen species (ROS) accompanied by the depletion of glutathione [11]. Also, the failures in the intracellular antioxidant systems, especially the reduction of the scavenger of lipid radical glutathione peroxidase 4 (GPX4), play important roles in the occurrence of ferroptosis [12]. Although the biological function of ferroptosis has not been completely illuminated, compelling evidence has shown a tight relationship between ferroptosis and oxidative stress [13, 14]. Many molecular events induced by oxidative stress, such as ischemic stroke and ionizing radiation, overlap with the development of ferroptosis, and ferroptosis, in turn, can be observed in various tissues and organs suffering from oxidative damage [5, 10, 15]. However, treatment with ROS inhibitors, such as N-acetylcysteine, could efficiently alleviate these lethal effects [16, 17]. Based on these findings, we speculated that the application of appropriate bioactive substances with the ability to facilitate ROS elimination and regulate ferroptosis may be an effective way to alleviate oxidative stress-associated intestinal injury.

Stilbenes are a series of natural compounds occurring in plant families that confer multiple benefits, such as antioxidant, anti-inflammatory, and cytoprotective actions [18–20]. Among them, pterostilbene, a 3,5-dimethylether derivative of resveratrol, has recently received particular attention due to its preferable bioavailability relative to the classical stilbenes, such as resveratrol [21]. The methoxy groups enhance the resistance of pterostilbene to phase II metabolism and elevate its lipophilicity. The latter further facilitates the membrane permeability of pterostilbene, making it easier to transport into enterocytes [22]. Importantly, many investigations using both *in vivo* and *in vitro* systems have revealed that pterostilbene has excellent efficiency in scavenging free radicals and inhibiting lipid peroxidation [23–25]. Our previous studies have also demonstrated that pterostilbene could effectively attenuate oxidative stress-associated intestinal damage in susceptible animals [19, 26]. However, whether pterostilbene could suppress ferroptosis under oxidative stress conditions has been not documented. Further, whether the protective effect of pterostilbene on intestinal health is associated with its regulation of ferroptosis remains uncertain.

Diquat is a nonselective and contact herbicide that transfers electrons to molecular oxygen and induces the production of superoxide anion and subsequently hydrogen peroxide (H_2_O_2_) in the mitochondria [27]. This property endows diquat with the potential to induce oxidative stress [19, 28, 29]. Here, we used a diquat-induced oxidative stress model with broiler chickens to explore whether pterostilbene supplementation could alleviate diquat-induced intestinal oxidative stress, barrier dysfunction, and ferroptosis and elucidate the possible mechanisms.

## 2. Materials and Methods

### 2.1. Animal, Diet, and Experimental Design

All animal procedures were approved by the Institutional Animal Care and Use Committee of Nanjing Agricultural University (SYXK-2017-0007). One hundred and ninety-two one-day-old male broiler chicks (Ross 308) were randomly divided into 4 groups: CON-SS group, PTS-SS group, CON-DQ group, and PTS-DQ group. Each treatment group comprised 6 replicates cage with 8 chickens per replicate. Broilers in the CON-SS and CON-DQ groups accepted a basal diet while those in the PTS-SS and PTS-DQ groups were fed a basal diet supplemented with 400 mg/kg pterostilbene (purity ≥ 99%; #537-42-8; BOC Sciences, Shirley, NY, USA). The feeding period was from day 1 to day 21. At 20 days of age, birds in the CON-DQ and PTS-DQ groups were injected intraperitoneally with 20 mg/kg body weight diquat (#6385-62-2; Sigma-Aldrich, St. Louis, MO), while those in the CON-SS and PTS-SS groups received the equivalent amount of sterile saline. The basal diet was formulated based on the NRC (1994) requirements, and its formulation is provided in Table S1. The supplementation level of pterostilbene in this research was determined based on the findings of our preliminary experiments (unpublished). Similarly, Zhang et al. [18] have demonstrated that 400 mg/kg pterostilbene effectively alleviated lipopolysaccharide-induced intestinal injury and immunological stress in broiler chickens. The injected dosage of diquat was chosen according to the previous studies [29, 30], in which administering diquat at 20 mg/kg body weight induced oxidative stress in broiler chickens. All birds were raised in an environmentally controlled room. The temperature was 33 ± 1°C during the first 3 days and then gradually decreased by 2 to 3°C per week until a temperature of 22 ± 1°C was achieved. A lighting schedule was set according to the recommendation of Ross Broiler Management Guide (2016). The feed and water were offered ad libitum.

### 2.2. Sample Collection

One broiler was randomly chosen from each replicate (cage) for sampling at 24 h postinjection. The blood samples collected from the neck vein were transferred into Na heparin vacutainers and centrifuged at 2,000 g for 10 min at 4°C to obtain the plasma samples. Afterward, the broilers were slaughtered by jugular vein exsanguination, and the jejunal tissue was harvested from the abdominal cavity immediately. For observation of morphology and apoptosis, approximately 1 cm of jejunal samples was taken from the middle of the jejunum and fixed with 4% paraformaldehyde. Subsequently, the jejunal mucosa from the remaining jejunum samples was scraped with sterile slides, frozen with liquid nitrogen, and stored at -80°C for further assessment.

### 2.3. Determination of Antioxidant-Related Parameters

The jejunal mucosa samples were weighed, homogenized with chilled sterile saline (1 : 4; weight/volume), and centrifugated at 4,000 g for 15 min at 4°C to obtain the supernatant. The activities of superoxide dismutase (SOD; #A001-1) and glutathione peroxidase (GPX; #A005-1) and the level of reduced glutathione (GSH; #A006-1) in plasma and jejunal supernatant and the malondialdehyde (MDA; #A003-1-2) concentration in jejunal supernatant were measured using the commercial kits purchased from Nanjing Jiancheng Institute of Bioengineering (Nanjing, Jiangsu, China). For measurement of H_2_O_2_ content, jejunal mucosa and plasma samples were homogenized with acetone followed by centrifugation at 8,000 g at 4°C. Then, the H_2_O_2_ level was detected using the titanium sulfate method in conformity with the manufacturer's descriptions (#bc3595; Solarbio; Beijing, China). Jejunal glutathione reductase (GR) activity was measured by reduced nicotinamide adenine dinucleotide phosphate- (NADPH-) dependent reduction of oxidized glutathione method as elucidated previously [26]. Jejunal thioredoxin reductase (TRXR) activity was tested using a colorimetric kit (#KA0883; Abnova, Beijing, China). Jejunal 4-hydroxy-2-nonenoic acid (4-HNE) level was measured by an enzyme-linked immunosorbent assay in accordance with the manufacturer's instruction (#E-EL-0128c; Elabscience; Wuhan, Hubei, China).

### 2.4. Measurement of Plasma Diamine Oxidase Activity and D-Lactate Content

Plasma diamine oxidase activity was measured by the method previously documented [31] with some modulation using a commercial kit (#A088; Nanjing Jiancheng Institute of Bioengineering). Nicotinamide adenine dinucleotide can be quantitatively oxidized by ammonia generated by diamine oxidase and monitored at 340 nm. Using the D-lactate Colorimetric Assay Kit (#AAT-13811; AAT Bioquest, Sunnyvale, CA, USA), we determined plasma D-lactate level. Briefly, D-lactate can be specifically oxidized by D-lactate hydrogenase, along with the generation of the proportional colorimetric product.

### 2.5. Jejunal Morphology Assay

The jejunal tissues were harvested and immersed in 4% paraformaldehyde overnight. Subsequently, the jejunal samples were dehydrated in a graded series of alcohol solutions, embedded in paraffin wax, and sectioned into 5 *μ*m pieces. The sections were deparaffinized, hydrated, dipped into hematoxylin buffer and eosin buffer (#G1120; Solarbio), and analyzed under a light microscopy (Nikon Eclipse 80i, Tokyo, Japan). Fifteen well-oriented villi of each sample were randomly selected to measure the villus height (VH), crypt depth (CD), and the ratio of villus height to crypt depth (VCR) of jejunum using the ImageJ software.

### 2.6. Jejunal Apoptosis Analysis

The apoptosis of jejunal tissues was evaluated using a TUNEL Apoptosis Detection Kit (#40308ES20; Yeasen; Shanghai, China). Briefly, paraffin section samples were dewaxed, rehydrated in ascending alcohol series (90, 80, and 70%), and permeabilized with proteinase K. After washing with PBS, the slices were exposed to equilibration buffer for 30 min followed by TdT reaction mixture for 1 h at 37°C in a dark humidified chamber. The 2-(4-amidinophenyl)-6-indolecarbamidine dihydrochloride (DAPI) was used to identify the nucleus. The sections were observed under a fluorescence microscope (Nikon Eclipse 50i, Tokyo, Japan) at ×100 magnification. Ten villi from each section were randomly selected by a blinded investigator to calculate the apoptotic rates using the ImageJ software.

### 2.7. Mitochondria Isolation and Determination of Mitochondrial ROS and Respiratory Complex Activity

For extraction of mitochondria, the jejunal mucosa samples were homogenized with a buffer (250 mmol/L sucrose, 3 mmol/L EDTA, and 1 mmol/L DTT; pH = 7.4) on ice. After centrifugation at 600 g for 15 min, the supernatant fraction was recentrifuged at 10,000 g for 10 min. Then, the pellet fraction was resuspended with the sucrose solution and centrifuged at 10,000 g for 10 min to collect the mitochondria-rich pellet for further analysis. Mitochondrial ROS was determined using a fluorescence probe dihydroethidium (DHE; #S0063; Beyotime, Shanghai, China) according to the protocols reported previously [19]. The activities of mitochondrial respiratory complexes (complex I, complex III, complex IV, and ATP synthase) were analyzed as previously described [32] with the corresponding kits obtained from Solarbo (#BC0510, #BC3240, #BC0940, and #BC1445).

### 2.8. Measurement of Superoxide Dismutase 2 (SOD2) Activity

The jejunal mucosa supernatant was prepared as described in Materials and Methods and Determination of Antioxidant-Related Parameters. Subsequently, the SOD2 activity of each sample was determined using a commercial kit (#S0103; Beyotime).

### 2.9. Mitochondrial DNA (mtDNA) Analysis

The total DNA was isolated from jejunal tissue using a DNA Extraction Kit (#9450; Takara; Dalian, Liaoning, China) as recommended in the manufacturer's guidelines. The levels of mtDNA and nuclear amplicons were quantified using qRT-PCR analysis by amplifying reduced form of nicotinamide adenine dinucleotide (NADH) dehydrogenase subunit 2 (forward: 5′-TCCCACCATTAACCGGCTTC-3′, reverse: 5′-AGTGGTATGCAAGTCGGAGG-3′) and 18S rRNA (forward: 5′-GTCTAAGTACACACGGGCGG-3′; reverse: 5′-TCCAAGTAACGGGAGGGGAG-3′), respectively. The relative amounts of mtDNA and nuclear DNA copy numbers were compared.

### 2.10. Determination of ATP Level

The ATP level in jejunal mucosa was measured by means of a luciferin/luciferase method with an ATP Bioluminescence Assay Kit (#S0026; Beyotime). Briefly, the jejunal mucosa samples were homogenized with a lysis buffer followed by centrifugation at 12,000 × g for 5 min at 4°C. After adding 90 *μ*L ATP testing buffer to 10 *μ*L of supernatant, the luminance was recorded by a fluorescence microplate reader (Tecan; Mannedorf, Switzerland). The protein content of each sample was measured by a Bradford Protein Assay Kit (#P0010; Beyotime) to normalize the ATP content.

### 2.11. Measurement of Iron Content

Jejunal mucosa samples were homogenized with sterile saline (1 : 4; weight/volume) on ice and centrifugated at 2,500 rpm for 10 min to collect the supernatant. Subsequently, iron content in jejunal mucosa supernatant was determined using an Iron Assay Kit (#BC4350-2-1, Solarbio) following the manufacturer's protocol. Briefly, ferric iron was reduced by reductants in an acidic buffer. Then, the iron (ferric iron and ferrous ion) reacted with double pyridine and generated a colorimetric product at the wavelength of 520 nm.

### 2.12. Total RNA Isolation and Gene Expression Detection

The total RNA from frozen jejunum samples was extracted with RNAiso Plus reagent (#9108; Takara). The determination of the quality and quantity of RNA was conducted based on the method in our previous publications [20, 29]. Then, 1 *μ*g RNA was reverse-transcribed to complementary DNA (cDNA) using a Hifair® III 1st Strand cDNA Synthesis Kit with gDNA eraser (#11139ES60; Yeasen). qRT-PCR was performed to detect the mRNA expression levels of occludin (*OCLN*), zona occludens 1 (*ZO-1*), ATP synthase alpha subunit (*ATP5A1*), ATP synthase beta polypeptide (*ATP5B*), nuclear respiratory factor 1 (*NRF1*), mitochondrial transcription factor A (*TFAM*), *GPX4*, solute carrier family 7 member 11 (*SLC7A11*), solute carrier family 11 member 2 (*SLC11A2*), long-chain acyl-CoA synthetase 4 (*ACSL4*), transferrin (*TF*), transferrin receptor (*TFRC*), ferritin heavy chain 1 (*FTH1*), ferritin light chain (*FTL*), heme oxygenase 1 (*HO1*), superoxide dismutase 1 (*SOD1*), *SOD2*, peroxiredoxin 1 (*PRDX1*), peroxiredoxin 3 (*PRDX3*), glutamatecysteine ligase modifier subunit (*GCLM*), glutamatecysteine ligase catalytic subunit (*GCLC*), and beta-actin (*β-actin*) using Hieff® qPCR SYBR Green Master Mix with High Rox (#11202ES03; Yeasen) and ABI StepOnePlus™ Real-Time PCR System (Applied Biosystems, Foster City, CA, USA). The reaction steps consisted of an initial denaturation at 95°C for 5 min and 40 cycles of 95°C for 10 s and 60°C for 30 s. The relative mRNA expression levels of the target genes were statistically analyzed using the 2^−*ΔΔ*Ct^ method, and *β-actin* was used as an internal normalization control. The list of qRT-PCR primers is included in Table S2.

### 2.13. Western Blot Analysis

Nuclear protein samples were isolated from jejunal mucosa by a Nuclear and Cytoplasmic Protein Extraction Kit (#P0027; Beyotime) with strict adherence to the manufacturer's protocol. The RIPA Lysis Buffer (#0010; Beyotime) with protease and phosphatase inhibitors was used to extract the total protein of jejunum. The protein concentrations were quantified by the Bradford method (#P0010; Beyotime). Approximately 25 *μ*g of protein in each lane was resolved by sodium dodecyl sulfate polyacrylamide (SDS-PAGE) gel and electrotransferred onto polyvinylidene difluoride (PVDF) membranes. The protein ladder was used as a molecular marker. After blocking with a QuickBlock™ Blocking Buffer for Western blot (#P0252; Beyotime), the PVDF membranes were washed with the Tris-buffered saline containing Tween-20 (TBST) and incubated with the primary antibodies against OCLN (1 : 3,000; #13409-1-AP; Proteintech; Rosemont, IL, USA), ZO-1 (1 : 1,000; #21773-1-AP; Proteintech), GPX4 (1 : 3,000; #67763-1-Ig; Proteintech), SLC7A11 (1 : 1,000; #A13685; ABclonal, Wuhan, Hubei, China), FTH1 (1 : 1,000; #A19544; ABclonal), ACSL4 (1 : 1,000; #A6826; ABclonal), NRF2 (1 : 1,000; #16396-1-AP; Proteintech), HO1(1 : 1,000; #27282-1-AP; Proteintech), SOD2 (1 : 5,000; #24147-1-AP; Proteintech), lamin B1 (1 : 5,000; #12987-1-AP; Proteintech), and *β*-actin (1 : 10,000; #60004-1-Ig; Proteintech) at 4°C overnight. Thereafter, the PVDF membranes were washed with TBST and probed with HRP-conjugated secondary antibodies (1 : 5,000; #SA00001-1; #SA00001-2; Proteintech) for 1.5 h at room temperature. The protein signals were monitored using Immobilon® ECL HRP Substrate (#WBULS0100; Millipore, Bedford, MA, USA). The bands were captured by a multi-imaging system (Tanon, Shanghai, China), and the results were quantified using the ImageJ software.

### 2.14. Statistical Analysis

The graphing and statistical analysis of the results was carried out using SPSS 22.0 (SPSS Inc., Chicago, IL, USA). All data are expressed as mean values with standard error. One-way analysis of variance and Tukey's post hoc test were performed to assess the significance of differences among multiple treatments. *P* < 0.05 was deemed statistically significant.

## 3. Results

### 3.1. Pterostilbene Alleviates Diquat-Induced Oxidative Stress in Broiler Chickens

**Figure 1:**
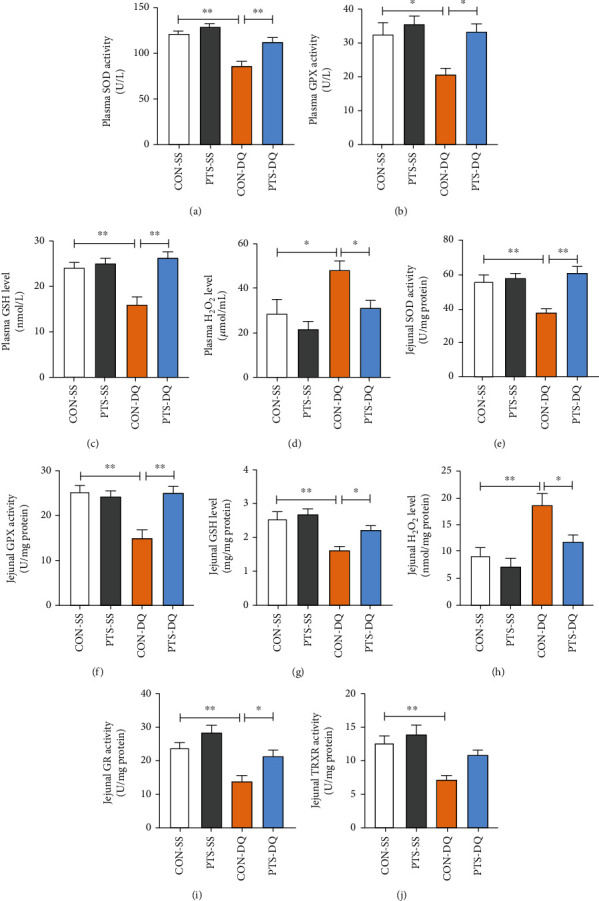
Pterostilbene mitigates diquat-induced oxidative stress in plasma and jejunum: (a) plasma SOD activity; (b) plasma GPX activity; (c) plasma GSH level; (d) plasma H_2_O_2_ level; (e) jejunal SOD activity; (f) jejunal GPX activity; (g) jejunal GSH level; (h) jejunal H_2_O_2_ level; (i) jejunal GR activity; (j) jejunal TRXR activity. Data are shown as mean ± standard error, *n* = 6/group; ^∗^*P* < 0.05 and ^∗∗^*P* < 0.01.

Many studies have successfully established *in vivo* oxidative stress models with diquat [29, 33]. In the present study, diquat-challenged broilers showed lower plasma SOD, GPX, and GSH levels but higher plasma H_2_O_2_ content than the control broilers (Figures 1(a)–1(d)), indicating the occurrence of systematic oxidative stress in diquat-challenged broilers. Similarly, diquat decreased the activities of SOD, GPX, GR, and TRXR and the level of GSH but increased H_2_O_2_ content in the jejunum when compared with the CON-SS group (Figures 1(e)–1(j)). By contrast, feeding with a pterostilbene-supplemented diet dramatically enhanced the activities of SOD and GPX and GSH content whereas inhibited the excessive accumulation of H_2_O_2_ in plasma and jejunum. Pterostilbene treatment also elevated jejunal GR activity of diquat-exposed broilers. These findings demonstrated the potential of pterostilbene to alleviate oxidative stress caused by diquat.

### 3.2. Pterostilbene Improves Intestinal Morphology and Barrier Function in Diquat-Challenged Broilers

**Figure 2:**
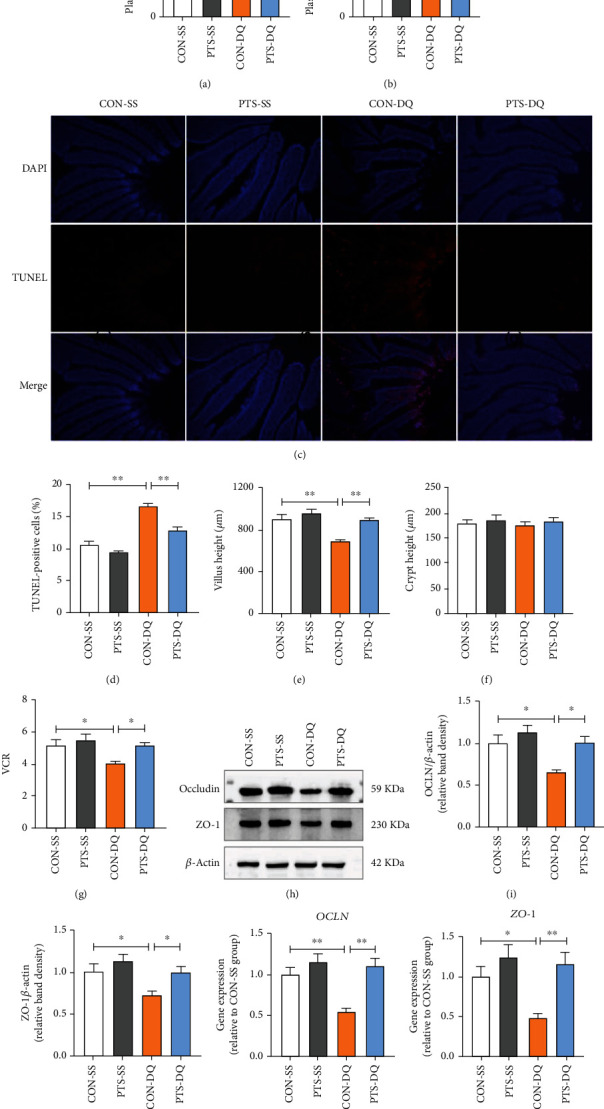
Effects of pterostilbene on intestinal permeability, jejunal apoptosis index, jejunal morphology and the expression of jejunal tight junction proteins in the diquat-challenged broilers. (a) Plasma DAO activity. (b) Plasma D-lactate level. (c) TUNEL analysis was used to identify the apoptotic cells in the jejunum. (d) The quantification of jejunal TUNEL-positive cells. (e) Jejunal villus height. (f) Jejunal crypt depth. (g) The ratio of villus height to crypt depth in the jejunum. (h–j) Western blot analysis was conducted to determine the protein expression of OCLN and ZO-1 in the jejunum. (k, l) qRT-PCR analysis was performed to detect the mRNA expression of *OCLN* and *ZO-1* in the jejunum. Data are shown as mean ± standard error, *n* = 6/group; ^∗^*P* < 0.05 and ^∗∗^*P* < 0.01.

Oxidative stress damages the proteins, lipids, and DNA in the enterocytes and impairs intestinal morphology and barrier integrity, leading to intestinal barrier dysfunction. To elucidate the protection of pterostilbene on diquat-induced intestinal injury, we monitored the changes in circulatory DAO activity and D-lactate level, two crucial parameters inflicting intestinal permeability and mucosa damage. Compared with the CON-SS broilers, broilers exposed to diquat showed obvious elevations in plasma DAO activity and D-lactate level (Figures 2(a) and 2(b)); however, these adverse effects were inhibited by supplementation with pterostilbene. In addition, diquat challenge significantly increased jejunal apoptosis rate and reduced jejunal VH and VCR (Figures 2(c)–2(g)). Conversely, pterostilbene administration dramatically decreased the number of jejunal TUNEL-positive cells and enhanced jejunal VH and VCR of diquat-challenged broilers.

Intestinal tight junctions are the determinants for intestinal function since they provide a physical intercellular barrier that restricts paracellular transport and regulates the selective permeability between epithelial cells [4]. Next, we explored the influence of pterostilbene on the expression of intestinal tight junction proteins. As indicated in Figures 2(h)–2(l), diquat-challenged broilers had lower mRNA and protein expression levels of OCLN and ZO-1 in the jejunum when compared with the CON-SS group. In contrast, administering pterostilbene significantly upregulated the mRNA and protein expression of jejunal OCLN and ZO-1 of diquat-challenged broilers. These data collectively indicated that pterostilbene mitigated diquat-induced mucosa damage and intestinal barrier dysfunction.

### 3.3. Pterostilbene Enhances Mitochondrial Function in the Jejunum of Diquat-Challenged Broilers

**Figure 3:**
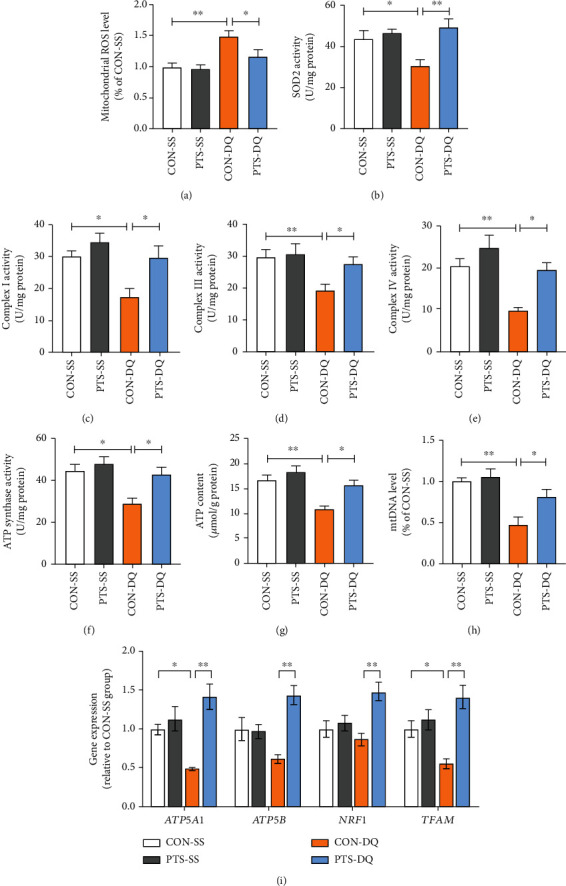
Effects of pterostilbene on mitochondrial redox status and function in the jejunum of diquat-challenged broilers. (a) Mitochondrial ROS in the jejunum was measured by a fluorescence probe DHE. (b) Jejunal SOD2 activity. (c–f) The jejunal activities of mitochondrial complexes I, III, and IV and ATP synthase. (g) Jejunal ATP level. (h) Jejunal mtDNA content. (i) qRT-PCR analysis was conducted to detect the expression of genes related to mitochondrial biogenesis in the jejunum. Data are shown as mean ± standard error, *n* = 6/group; ^∗^*P* < 0.05 and ^∗∗^*P* < 0.01.

Mitochondria are both the principal generators and targets of ROS [34]. Mitochondrial dysfunction is inseparably linked to oxidative stress. To verify whether pterostilbene could attenuate diquat-induced mitochondrial injury in broilers, multiple markers of mitochondrial redox status and function were examined. Here, diquat challenge induced a remarkable increase in mitochondrial ROS but a decline in SOD2 activity in the jejunum (Figures 3(a) and 3(b)). Treatment with diquat also significantly reduced the activities of mitochondrial complexes I, III, and IV and ATP synthase and the levels of ATP and mtDNA in the jejunum (Figures 3(c)–3(h)). In contrast, these negative effects caused by diquat were alleviated by pterostilbene administration. Moreover, we investigated the effects of pterostilbene on the expression of genes related to mitochondrial biogenesis in the jejunum. Compared with the control broilers, the mRNA abundance of *ATP5A1* and *TFAM* in the jejunum of the CON-DQ broilers was significantly repressed but recovered following pterostilbene supplementation (Figure 3(i)). Also, pterostilbene upregulated the mRNA abundance of *ATP5B* and *NRF1* in the jejunum of diquat-challenged broilers. These findings indicated that pterostilbene improved mitochondrial dysfunction in the jejunum of diquat-challenged broilers.

### 3.4. Pterostilbene Inhibits Diquat-Induced Ferroptosis in the Jejunum of Broilers

**Figure 4:**
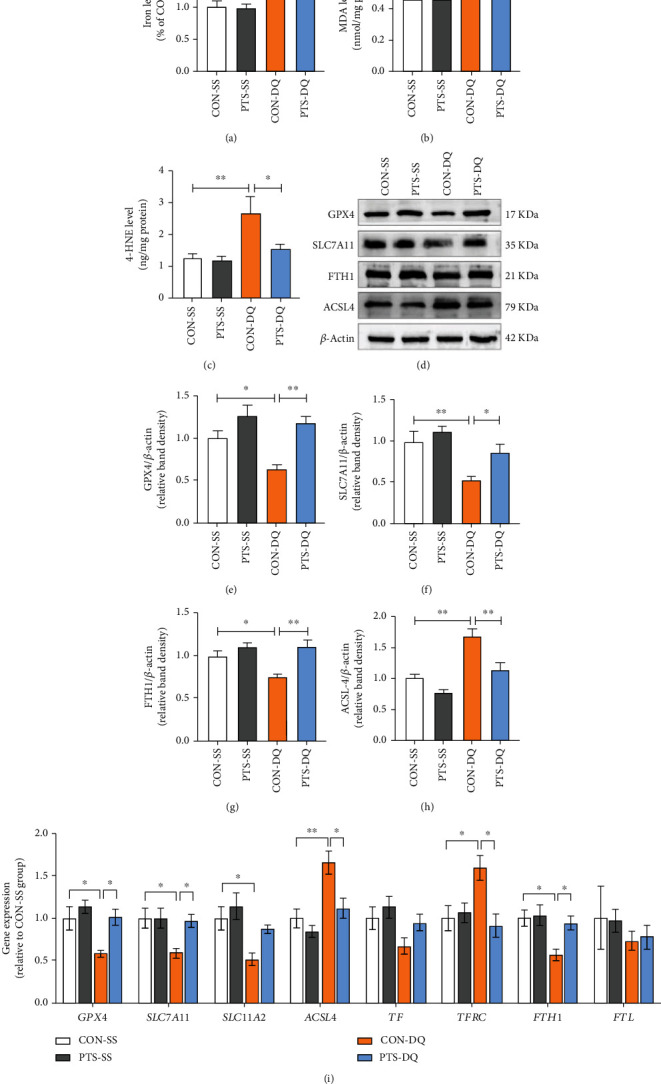
Pterostilbene suppresses jejunal ferroptosis of diquat-challenged broilers. (a) Jejunal iron content. (b) Jejunal MDA level. (c) Jejunal 4-HNE level. (d–h) Western blot analysis was conducted to determine the protein levels of GPX4, SLC7A11, FTH1, and ACSL4 in the jejunum. (i) qRT-PCR analysis was conducted to detect the expression of genes related to ferroptosis in the jejunum. Data are shown as mean ± standard error, *n* = 6/group; ^∗^*P* < 0.05 and ^∗∗^*P* < 0.01.

To illuminate whether ferroptosis was involved in the diquat-induced intestinal injury, multiple ferroptosis indicators were measured. Compared with the control broilers, the diquat-challenged broilers exhibited prominently higher levels of iron, MDA, and 4-HNE in the jejunum (Figures 4(a)–4(c)), suggesting that diquat exposure elicited ferroptosis of jejunum. After pterostilbene treatment, the aberrant accumulation of iron, MDA, and 4-HNE in the jejunum of diquat-challenged broilers was remarkably restrained. To further dissect how pterostilbene regulated ferroptosis under oxidative stress conditions, we detected ferroptosis-related signaling pathways. As indicated in Figures 4(d)–4(i), the mRNA and protein levels of ferroptosis-negative regulators GPX4, SLC7A11, and FTH1 in the jejunum were reduced by diquat exposure, while the mRNA and protein levels of the positive regulator ACSL4 exhibited an opposite trend. In addition, diquat considerably downregulated the mRNA abundance of *SLC11A2* but upregulated the mRNA abundance of *TFRC* in the jejunum. In contrast, pterostilbene treatment induced the mRNA and protein expressions of GPX4, SLC7A11, and FTH1 but inhibited the mRNA and protein expressions of ACSL4 and the mRNA abundance of *TFRC* in the jejunum of diquat-exposed broilers. These data suggested that pterostilbene mitigated jejunal ferroptosis of diquat-challenged broilers.

### 3.5. Pterostilbene Activates NRF2 Signaling Pathway in the Jejunum of the Diquat-Challenged Broilers

**Figure 5:**
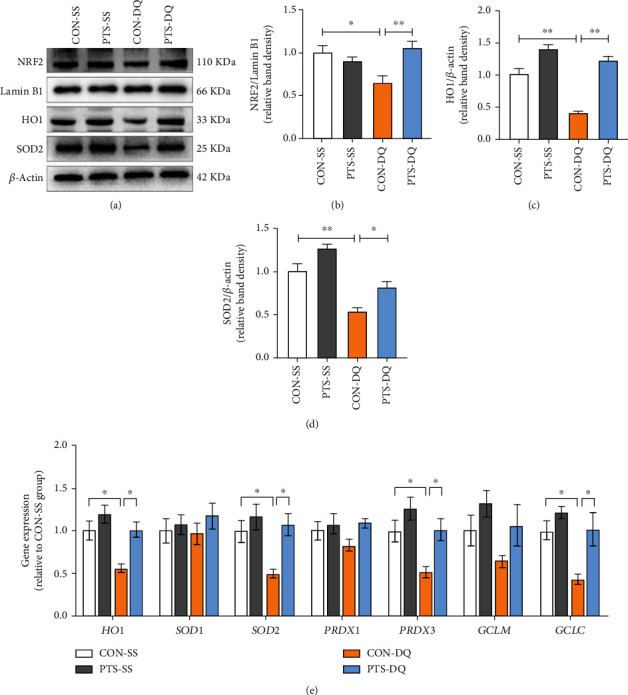
Pterostilbene activates NRF2 signals in the jejunum of diquat-challenged broilers. (a–d) Western blot analysis was conducted to determine the protein levels of nuclear NRF2, HO1, and SOD2 in the jejunum. (e) qRT-PCR analysis was conducted to detect the mRNA expression of NRF2 targets in the jejunum. Data are shown as mean ± standard error, *n* = 6/group; ^∗^*P* < 0.05 and ^∗∗^*P* < 0.01.

The NRF2 is not only a master antioxidative transcription factor but also a negative regulator of ferroptosis [35]. We next detected the expression levels of NRF2 signaling pathways to reveal the potential mechanisms that pterostilbene alleviated diquat-induced intestinal oxidative stress and ferroptosis. As illustrated in Figures 5(a)–5(e), diquat exposure remarkably inhibited the NRF2 protein expression in the nucleus and decreased its downstream targets HO1 and SOD2 protein levels in the jejunum compared with the control subjects. Similar to the above results, the mRNA abundance of *HO1*, *SOD2*, *PRDX3*, and *GCLC* in the jejunum of the diquat-exposed broilers was significantly downregulated. On the contrary, pterostilbene supplementation promoted the translocation of NRF2 from the cytoplasm to the nucleus and upregulated the mRNA and protein levels of HO1 and SOD2 in the jejunum of broilers exposed to diquat. Pterostilbene also improved diquat-induced decreases in the mRNA abundance of *PRDX3* and *GCLC* in the jejunum of broilers. Taken together, pterostilbene induced the activation of NRF2 signals in the jejunum of diquat-exposed broilers.

## 4. Discussion

Several studies have revealed that diquat is an effective chemical agent to trigger oxidative stress owing to its ability to produce ROS continuously by cyclic reduction-oxidation processes [19, 27, 28, 36]. In the present study, diquat decreased jejunal activities of SOD, GPX, GR, and TRXR and the level of GSH whereas increased jejunal H_2_O_2_ content indicating that there was severe oxidative stress in the jejunum. Interestingly, plasma DAO activity and D-lactate content of broilers were also raised after diquat treatment. D-Lactate is a metabolite of intestinal bacteria that traverses the intestinal barrier when the intestinal permeability was aberrantly increased [37]. DAO is an intracellular enzyme localized in the small intestinal epithelia, and it enters the circulation through the impaired intestinal barrier [38]. Accumulating evidence with broilers has demonstrated that the increases in circulating D-lactate and DAO were linked with gut mucosa damage and barrier dysfunction [37, 39]. These findings collectively suggested that diquat challenge induced intestinal oxidative stress and disrupted mucosal barrier function of broilers.

OCLN and ZO1 are the crucial components of intestinal tight junctions. OCLN strengthens barrier integrity by interacting with multiple intracellular signaling pathways and further restricts intestinal permeability to low molecular mass molecules [40]. Loss of OCLN expression is closely related to barrier dysfunction in various diseases, such as inflammation and ischemia [41]. ZO-1 is obligate for the link of transmembrane junctional proteins to the actomyosin cytoskeleton and some cytoplasmic regulatory proteins [42]. Here, diquat significantly decreased the mRNA and protein levels of OCLN and ZO1, this may explain the compromised intestinal barrier function of the diquat-exposed broilers.

In addition, the elevated cell death in the jejunum constitutes another potential mechanism for the diquat-induced intestinal barrier dysfunction of broilers. Apoptosis is a programmed cell death process that eliminates damaged or unwanted cells and modulates cellular homeostasis [43]. We observed that diquat increased the numbers of apoptotic cells in the jejunum which may be attributed to the damaged mitochondria [19, 36]. In consistence with our previous findings, diquat disturbed the redox balance of mitochondria, showing increased mitochondrial ROS level and decreased SOD2 activity. These negative alterations further suppressed electron transport chain efficiency and hindered mitochondrial biogenesis, leading to ATP deficiency and apoptosis. Interestingly, diquat induced not only apoptosis but also ferroptosis, an emerging form of cell death manifested as iron-dependent lipid peroxidation [11]. 4-HNE and MDA are the two major secondary productions of lipid peroxidation generated by the decomposition of oxidized polyunsaturated fatty acids (PUFAs), which have been considered as the ultimate executor of ferroptosis [44, 45]. Therefore, diquat-induced increases in 4-HNE and MDA levels in the jejunum, together with iron accumulation, confirmed the occurrence of ferroptosis in intestinal cells. Furthermore, diquat-challenged broilers showed lower VH and VCR in the jejunum than the control subjects, implying an impaired absorptive capacity of the small intestine. Taken together, the defects in intestinal redox balance, barrier function, and morphology observed above may account for the body weight loss of the diquat-challenged broilers described in a previous study [29].

Many investigations have revealed the potential of pterostilbene for intestinal health in animal models [18, 26, 46]. Here, we demonstrated that diquat-induced intestinal barrier dysfunction was efficiently recovered with the evidence of the decreased circulating DAO activity and D-lactate content, increased tight junction proteins, and improved jejunal morphology. In addition, pterostilbene restored the redox balance of plasma and jejunum in diquat-exposed broilers, which is possibly associated with its superior antioxidative activity [18, 26, 47, 48]. Owing to the existence of 4′-hydroxyl group, pterostilbene can directly eliminate endogenous free radicals, including superoxide, hydroxyl, and H_2_O_2_ [24]. Meanwhile, pterostilbene is able to activate antioxidant defense systems to mitigate oxidative damage [49]. SOD is an important detoxification enzyme that catalyzes the highly reactive and unstable superoxide anion to less hazardous hydrogen peroxide at diffusion limiting rates, and the latter can be further eliminated by either GPX or catalase [50]. Studies have demonstrated that pterostilbene enhanced the activities of SOD and GPX in a variety of adverse circumstances, such as intestinal ischemia reperfusion injury [47] and ulcerative colitis [48]. Consistently, the plasma and jejunal activities of SOD and GPX of diquat-exposed broilers in this study were remarkably increased by pterostilbene. These findings identified the strong antioxidant capacity of pterostilbene and explained the decreases in circulatory and jejunal H_2_O_2_ levels of broilers fed a pterostilbene-supplemented diet.

Another mechanism whereby pterostilbene mitigates diquat-induced intestinal injury is probably related to its robust effect against ferroptosis. In this study, pterostilbene reversed diquat-induced ferroptosis showing reduced iron content and lipid peroxidation products (4-HNE and MDA) and elevated GSH level in the jejunum. The protective mechanisms by which occurred are complicated and probably refer to two aspects. First, pterostilbene maintained the iron homeostasis of diquat-exposed broilers. Iron is a main redox-active toxicant that generates ROS through the Fenton reaction [11]. Normally, most circulating iron is bound to the TF, captured by transferrin receptor 1 on the cellular membrane, and delivered into cells. When intracellular iron was excessive, it can be sequestered as ferritin [11]. In this study, pterostilbene upregulated the mRNA and protein expression of TFH1 but downregulated *TFRC* mRNA of diquat-challenged broilers implying that pterostilbene prevented diquat-induced iron accumulation by suppressing iron uptake. Second, pterostilbene protected the intestine against diquat-induced lipid peroxidation. Owing to its preferable lipophilicity and membrane permeability, pterostilbene can detoxify membrane lipid hydroperoxides [24]. In addition, ACSL4, a key enzyme that increases ferroptosis sensitivity by incorporating PUFAs into the cellular phospholipids [51], was downregulated by pterostilbene. Moreover, pterostilbene regulated GSH/GPX4 pathway in the jejunum of diquat-exposed broilers. GSH is a thiol-containing tripeptide and acts as a reductant in intracellular antioxidant defense [52]. Importantly, GSH serves as an indispensable cofactor of GPX4, the sole peroxidase reducing the cytotoxic lipid peroxides to nontoxic lipid alcohols within biomembranes [52]. Intracellular GSH level can be modulated by GR-mediated reduction reaction. System XC^−^, especially SLC7A11, also plays a vital role in GSH synthesis [44]. In this trial, pterostilbene improved diquat-induced GSH depletion by enhancing GR activity and upregulating the mRNA and protein levels of SLC7A1. Further, pterostilbene promoted the mRNA and protein expression of GPX4 of diquat-exposed broilers. These data suggested the powerful potential of pterostilbene to inhibit diquat-induced lipid peroxidation.

Recent studies have demonstrated that pterostilbene is a potent activator of NRF2 [36, 39]. Pterostilbene inhibits the protein-protein interaction of NRF2-Kelch-like ECH-associated protein 1 (KEAP1) complex to facilitate the nuclear import of NRF2 and subsequently trigger a battery of cytoprotective genes [49]. Similarly, pterostilbene incurred an obvious increase in the nuclear accumulation of NRF2 protein in the jejunum of diquat-exposed broilers which further induced the expression of HO1, SOD2, and PRDX3, to counteract diquat-induced intestinal oxidative stress. SOD2 and PRDX3 are key mitochondrial antioxidant proteins. Thus, the increased *SOD2* and *PRDX3* mRNA may promote the elimination of mitochondrial ROS and consequently alleviate diquat-induced mitochondrial dysfunction. Interestingly, NRF2 can also regulate ferroptosis since NRF2 target genes are involved in the modulation of lipid peroxides and iron metabolism [35, 53]. Kwak et al. [54] have pointed out that NRF2-deficient mice had a lower mRNA level of *FTH1* than the NRF2-wild-type mice. In HEK293T cells, KEAP1 knockdown-induced nuclear translocation of NRF2 inhibited iron-induced ferroptosis by upregulating the protein expression of GPX4 and SLC7A11 [55]. Therefore, pterostilbene-mediated activation of NRF2 in this study may explain the changes in genes and proteins associated with iron metabolism and the reduced ferroptosis of diquat-exposed broilers.

## 5. Conclusions

The present investigation identifies pterostilbene as a promising candidate for diquat-induced intestinal injury of broiler chickens that regulates redox homeostasis, inhibits jejunal ferroptosis, and further improves jejunal morphology, mitochondrial function, and intestinal barrier defense. These findings may offer promising strategies for the treatment and prevention of oxidative stress and resultant intestinal injury.

## Data Availability

The data used to support the findings of the present study are available from the corresponding authors upon request.

## Supplementary Materials

Supplementary Materials Table S1: composition and calculated nutrient levels of basal diet. Table S2: sequences of primers for qRT-PCR.
