# Supplementary material for: Pterostilbene Confers Protection against Diquat-Induced Intestinal Damage with Potential Regulation of Redox Status and Ferroptosis in Broiler Chickens
Source: Oxid Med Cell Longev. 2023 Jan 24;2023:8258354. doi: 10.1155/2023/8258354 (PMC9889155; doi:10.1155/2023/8258354)
Supplement: Supplementary Materials — Table S1: composition and calculated nutrient levels of basal diet. Table S2: sequences of primers for qRT-PCR. [file 8258354.f1.docx]

**Table S1**. Composition and calculated nutrient levels of basal diet

| Items | Content | Items | Content |
| --- | --- | --- | --- |
| Ingredients (%) |  | Calculated nutrient levels (%)^2^ |  |
| Maize | 57.00 | Metabolizable energy (MJ/kg) | 12.53 |
| Soybean meal | 31.00 | Crude protein | 20.90 |
| Maize gluten meal | 3.90 | Calcium | 0.99 |
| Soybean oil | 3.10 | Available phosphorus | 0.43 |
| Limestone | 1.30 | Lysine | 1.05 |
| Dicalcium phosphate | 1.80 | Methionine | 0.51 |
| _L_-Lysine (78%) | 0.15 | Methionine + cystine | 0.85 |
| _DL_-Methionine (98%) | 0.15 |  |  |
| Sodium chloride | 0.30 |  |  |
| Premix^1^ | 1.00 |  |  |
| Zeolite | 0.30 |  |  |
| Total | 100.00 |  |  |

^1^Premix provided per kilogram of diet: transretinyl acetate, 24 mg; cholecalciferol, 6 mg; all-rac-α-tocopherol acetate, 20 mg; menadione, 1.3 mg; thiamin, 2.2 mg; riboflavin, 8 mg; nicotinamide, 40 mg; choline chloride, 400 mg; calcium pantothenate, 10 mg; pyridoxine·HCl, 4 mg; biotin, 0.04 mg; folic acid, 1 mg; vitamin B_12_ (cobalamin), 0.013 mg; Fe (from ferrous sulfate), 80 mg; Cu (from copper sulfate), 8.0 mg; Mn (from manganese sulfate), 110 mg; Zn (from zinc oxide), 65 mg; I (from calcium iodate), 1.1 mg; Se (from sodium selenite), 0.3 mg.

^2^The nutrient levels were as fed basis.

**Table S2** Sequences of primers for quantitative real time PCR

| Gene | Gene Bank ID | Primer sequence | Length |
| --- | --- | --- | --- |
| *ACSL4* | XM_040670613.1 | TTGCTCGCACTTGAGTCTGT | 128 |
|  |  | TTGCTCGCACTTGAGTCTGT |  |
| *ATP5A1* | AF332870.1 | GTCTGATTTGGATGCTGCCAC | 149 |
|  |  | CCAGCTTGTCCAGGTGACTTTT |  |
| *ATP5B* | NM_001031391.3 | AAGGCTCCATCACTTCGGTG | 183 |
|  |  | TGTTGGGGTCCATGATTCGG |  |
| *FTH1* | NM_205086.2 | GTCGTATCCACCGCATCTCT | 182 |
|  |  | GTCGTATCCACCGCATCTCT |  |
| *FTL* | NM_204383.2 | CAGACCCTCATAGTGCCAGC | 109 |
|  |  | GAACAATCAAGCCTGCGAGC |  |
| *GCLC* | XM_419910.5 | GGACGCTATGGGGTTTGGAA | 122 |
|  |  | AGGCCATCACAATGGGACAG |  |
| *GCLM* | NM_001007953.1 | GTGCTGAGTCACGGTGTCG | 193 |
|  |  | TGTTTTCTGAATGCAGTCCCG |  |
| *GPX4* | NM_001346448.2 | GGGGAATGCCATCAAGTGGA | 84 |
|  |  | TCCTCCATTGGGCTGTACCT |  |
| *HO1* | NM_205344.1 | GTCGTTGGCAAGAAGCATCC | 106 |
|  |  | GGGCCTTTTGGGCGATTTTC |  |
| *NRF1* | NM_001030646.1 | GCATTGAGTCTCTCTCCGCT | 104 |
|  |  | TGGTGCTACAAAGACGCACT |  |
| *OCLN* | NM_205128.1 | TGAATGCACCCACTGAGTGTT | 99 |
|  |  | CCAGAGGTGTGGGCCTTAC |  |
| *PRDX1* | XM_040674880.1 | GCTCACACTGGAAATCGTGG | 151 |
|  |  | ATTCATTTTGCCCAAGCCGC |  |
| *PRDX3* | NM_001271932.2 | AACAAGGTGGTTTGGGCACT | 182 |
|  |  | AACAGAACGGCCAACAGGAA |  |
| *SLC7A11* | XM_040670528.1 | ATGTTCTTCGTTGCCTCCCG | 198 |
|  |  | ATGTTCTTCGTTGCCTCCCG |  |
| *SLC11A2* | [DQ848155.1](https://www.ncbi.nlm.nih.gov/entrez/viewer.fcgi?db=nucleotide&id=112820475) | TCGCAGCAGTAATCGGACTC | 92 |
|  |  | GATCAGACACAGCCACGTCA |  |
| *SOD1* | NM_205064.1 | GGCAATGTGACTGCAAAGGG | 133 |
|  |  | CCCCTCTACCCAGGTCATCA |  |
| *SOD2* | NM_204211.1 | ACTGTTGTGCGACAAAGGGA | 143 |
|  |  | CACAAAGTGTGCGTTTCCACT |  |
| *TF* | NM_205304.2 | CAACCTCAGGGACCTCACAC | 167 |
|  |  | TGGGCTTCAGCTTGTATGGG |  |
| *TFAM* | NM_204100.1 | GCAGCTGTTCAGGGGCTG | 124 |
|  |  | CTGGTTGTCCCTCAGGAAGC |  |
| *TFRC* | NM_205256.2 | TGGAGACTCCTGATGCTATCG | 120 |
|  |  | GTGAAGCCACGACCTTCTGT |  |
| *ZO-1* | XM_413773.4 | TGTAGCCACAGCAAGAGGTG | 98 |
|  |  | CTGGAATGGCTCCTTGTGGT |  |
| *β-actin* | NM_204305.1 | CCCCCATGTTTGTGATGGGT | 162 |
|  |  | TGATGGCATGGACAGTGGTC |  |

*ACSL4*, long-chain acyl-CoA synthetase, *ATP5A1*, ATP synthase alpha subunit, *ATP5B*, ATP synthase beta polypeptide, *FTH1* ferritin heavy chain 1, *FTL*, ferritin light chain, *GCLC*, glutamatecysteine ligase catalytic subunit, *GCLM*, glutamatecysteine ligase modifier subunit, *GPX4*, glutathione peroxidases 4, *HO1*, heme oxygenase 1, *NRF1*, nuclear respiratory factor 1, *OCLN*, occludin, *PRDX1*, peroxiredoxin 1, *PRDX3*, peroxiredoxin 3, *SLC7A11*, solute carrier family 7 member 11, *SLC11A2*, solute carrier family 11 member 2, *SOD1*, superoxide dismutase 1, *SOD2*, superoxide dismutase 2, *TF*, transferrin, *TFAM*, mitochondrial transcription factor A, *TFRC*, transferrin receptor, *ZO-1*, zona occludens 1, *β-actin*, beta actin.
